# Repeated Hemolytic Streptococcal Infections in Soft Tissue Eosinophilic Granulomatosis (Kimura’s Disease): A Case Report

**DOI:** 10.7759/cureus.57811

**Published:** 2024-04-08

**Authors:** Miwako Togawa, Takushi Ikeda, Katsuya Konishi, Ryo Ichibayashi

**Affiliations:** 1 Department of Emergency Medicine, Toho University Sakura Medical Center, Chiba, JPN; 2 Department of Cardiovascular Medicine, Toho University Sakura Medical Center, Chiba, JPN; 3 Department of Dermatology, Toho University Sakura Medical Center, Chiba, JPN

**Keywords:** sepsis, infection, lymphadenopathy, group g streptococcus (ggs), kimura’s disease

## Abstract

Soft tissue eosinophilic granulomatosis (Kimura's disease) is an eosinophilic granuloma with a proliferation of lymphoid follicles in the subcutaneous soft tissue. Although no established treatment exists, it is considered a disease with a good prognosis. On the other hand, bacteremia caused by group G hemolytic streptococcus (GGS) is said to be caused by chronic local lymph abnormalities and is likely to recur.

We present the case of a 41-year-old Japanese man. He had a history of treatment for Kimura's disease and sepsis due to hemolytic streptococcus and came to our hospital with a chief complaint of fever. His blood culture revealed hemolytic streptococcus, and he was admitted to the hospital.

Kimura's disease involves the proliferation of lymphoid follicles, so when blood cultures repeatedly turn positive, it is important to treat the patient with a GGS infection in mind.

## Introduction

Soft tissue eosinophilic granulomatosis (Kimura’s disease) is an eosinophilic granuloma with a proliferation of lymphoid follicles in the subcutaneous soft tissue. The course is gradual, and masses often form in the head and neck region, especially around the parotid glands [1]. The mass is painless and has unclear borders. Blood tests are characterized by a marked increase in peripheral eosinophils and elevated serum IgE [2]. A definitive diagnosis requires histological examination to demonstrate marked proliferation of lymphocytes, formation of lymphoid follicles, and infiltration of eosinophils in the subcutaneous soft tissues and lymph nodes [3].

Although it has been suggested that immune/allergic reactions are involved in the pathogenesis of this disease, it is still unclear [1]. Treatment includes drugs, surgery, and radiation therapy, but no clear treatment method has been established [3]. Oral steroid therapy may have the effect of reducing the tumor mass, but there are many cases of repeated recurrence. For this reason, the side effects of long-term oral steroid use are avoided, and symptoms are often controlled by repeating short-term oral steroid use [4].

Although it is not fully understood, as described above, Kimura's disease is said to have a good prognosis. However, we have experienced a case in which a patient with Kimura's disease was repeatedly infected with streptococcus, leading to sepsis. There have been no reports of repeated streptococcal infections in Kimura disease. We report on its clinical characteristics with a review of the literature.

## Case presentation

The patient was a 41-year-old Japanese man. His past medical history included chronic hepatitis C, Kimura's disease, thyroid abscess (the causative agent was Streptococcus constellatus), and necrosis of the femoral head. Kimura's disease was diagnosed four years ago, and he took oral steroids for a year and a half. During this time, the patient had a history of treatment for left thyroid abscess and femoral head necrosis. Regular oral medications were duloxetine and loxoprofen.

**Figure 1:**
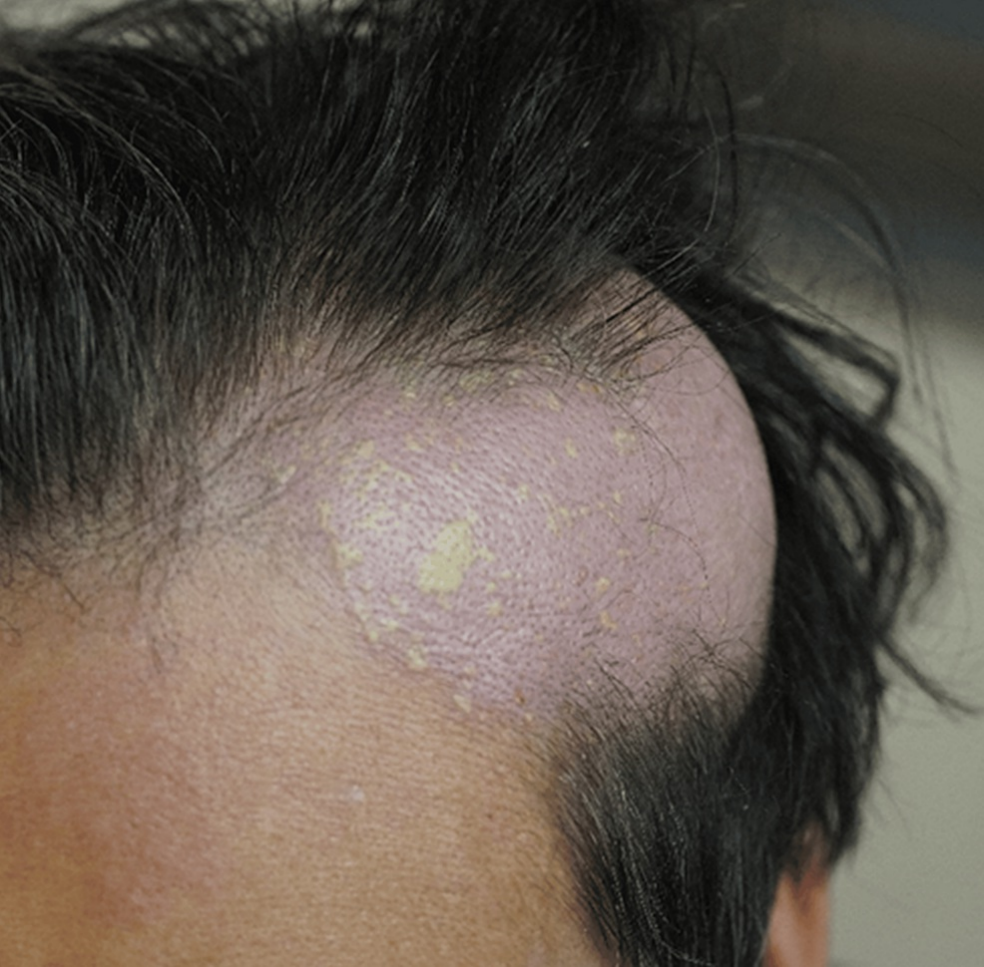
Photo of mass on the left side of the head The subcutaneous mass had a clear border and a sensation of heat and swelling. Pigmentation is observed on the surface of the subcutaneous mass.

He was transported by ambulance due to a fever of 40 degrees Celsius accompanied by chills and shivering. At the time of admission, his consciousness was E4V5M6 on the Glasgow Coma Scale (GCS), body temperature 39.8 °C, respiratory rate 20/min, pulse 123/min, blood pressure 131/72 mmHg, and oxygen saturation (SpO2) 98% (room air). A physical exam revealed a gently raised, elastic, subcutaneous mass on the left side of his head, accompanied by alopecia consistent with the lesion. The skin of the mass on the left side of the head showed a feeling of warmth and slight redness (Figure 1).

Cervical lymph node swelling was observed, but no tenderness was noted. He had tattoos on his chest and abdomen. Other findings included pain during exercise in the hip and knee joints. Blood tests revealed increased white blood cells and eosinophil percentage, with CRP 0.88 mg/dL and WBC 19370/μL (Eosino 30.5%) (Table 1).

**Table 1:** Laboratory results CRP: C-reactive protein, TP: total protein, Alb: albumin, AST: aspartate aminotransferase, ALT: alanine aminotransferase, LDH: lactate dehydrogenase, γ-GTP: γ-glutamyl transpeptidase, T-Bil: total bilirubin, BUN: blood urea nitrogen, eGFR: estimated glomerular filtration, HbA1c: hemoglobin A1c, WBC: white blood cell, Neut: neutrophil, Lymp: lymphocyte, Mono: monocyte, Eosino: eosinophil, Baso: basophil, RBC: red blood cell, Hb: hemoglobin, Ht: hematocrit, Plt: platelet

Test	Result	Unit	Reference range
CRP	0.88	mg/dL	<0.3
TP	7.5	g/dL	6.7-8.3
Alb	4.2	g/dL	3.8-5.2
AST	31	IU/L	10-40
ALT	42	IU/L	5-45
LDH	293	U/L	124-222
γ-GTP	39	IU/L	<30
T-Bil	1.1	mg/dL	0.2-1.2
BUN	14.5	mg/dL	8.0-20.0
Creatinine	1.16	mg/dL	0.47-0.79
eGFR	57	mL/min/1.73m^2^	>60
Sodium	139	mEq/L	137-147
Potassium	3.4	mEq/L	3.5-5.0
Chlorine	107	mEq/L	98-108
Glucose	110	mg/dL	70-109
HbA1c	5.7	％	4.7-6.2
WBC	19370	/μL	3300-9000
Neut	64	％	40-70
Lymp	6.0	％	20-50
Mono	0.5	％	2.0-9.0
Eosino	30.5	％	1.0-6.0
Baso	1.0	％	0-2.0
RBC	451	×10^4^/μL	430-570
Hb	14.4	g/dL	13.5-17.5
Ht	39.7	44.9%	39.7-52.4
Plt	17.4	10^4^/μL	14-34

**Figure 2:**
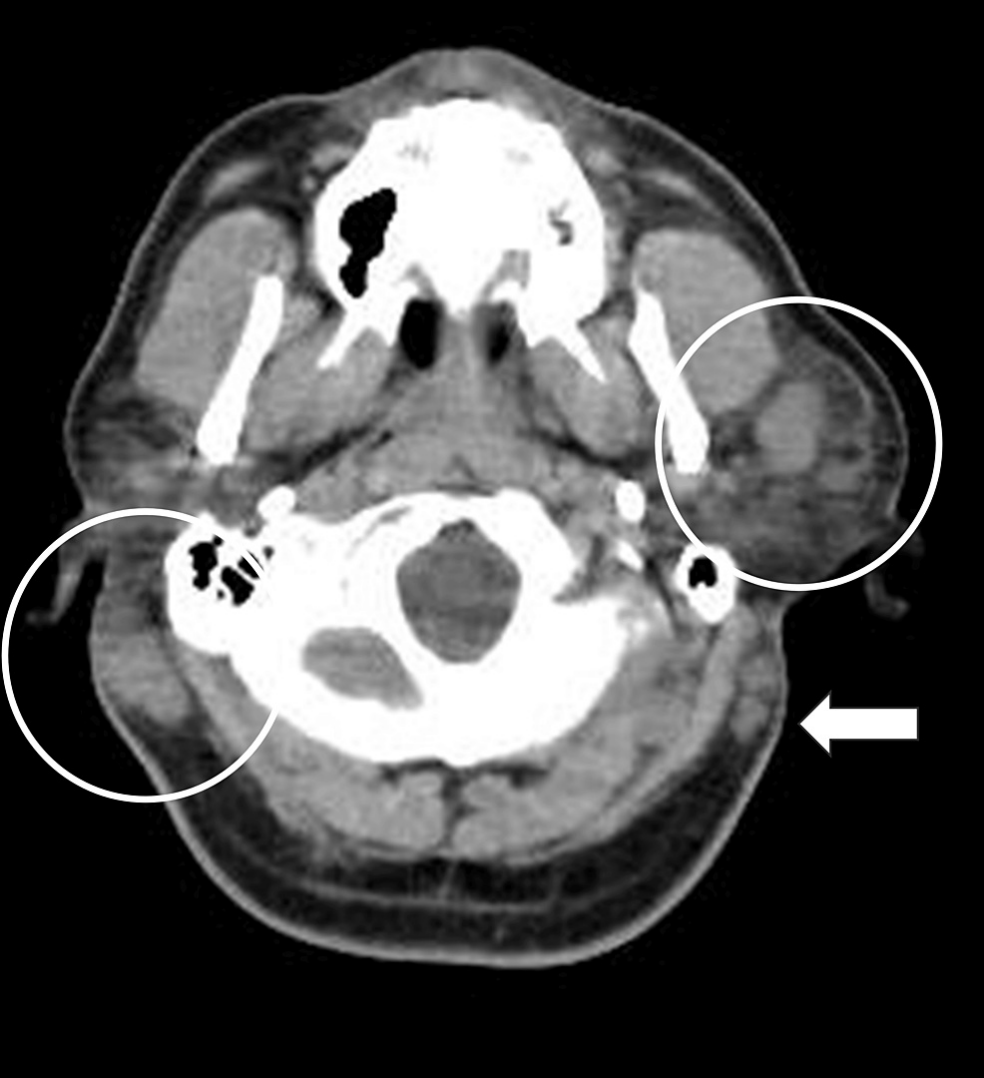
Cervical CT An infiltrative lesion with ill-defined borders has formed beneath the skin adjacent to the parotid gland (white circle). Enlarged lymph nodes are seen in the surrounding area (white arrows).

The electrocardiogram showed sinus tachycardia. Chest X-ray showed a cardiothoracic ratio of 0.48 and no abnormal findings. A whole-body CT scan was performed to search for infection foci causing the fever. A subcutaneous mass in the left frontal and parietal regions and parotid gland swelling were observed. Swollen lymph nodes were observed in the bilateral neck and supraclavicular fossae (Figure 2).

These were judged to be findings of Kimura's disease. A whole-body CT scan failed to identify a source of infection. Since the CRP level was slightly increased, antipyretics were prescribed, and the patient was instructed to go home. The patient revisited our hospital because the fever of 39 °C continued the next day. A re-examination of the blood test revealed that the CRP level was elevated at 22.34 mg/dL. Urine and cerebrospinal fluid findings were normal. A rapid antigen test for group A beta-hemolytic streptococcus (GAS) on a throat swab was positive. The patient was diagnosed with a GAS infection and was admitted to the hospital for treatment. After admission, two sets of Streptococcus dysgalactiae subsp. equisimilis (SDSE) were detected in blood cultures. A cardiac ultrasound revealed no vegetation on the valves. Antibiotics were sulbactam/ampicillin (SBT/ABPC) 9 g/day. On the fourth day of hospitalization, CRP decreased to 3.72 mg/dL. Blood cultures after the fourth day of hospitalization were negative. Although a mass was observed in the frontal region, the redness and heat sensation disappeared as the inflammatory response decreased. Therefore, cellulitis at the same site was considered the focus of infection. SBT/ABPC was continued, and the patient was discharged on the eighth day of hospitalization. After being discharged from the hospital, he was given oral amoxicillin (AMPC) for seven days. Blood tests on the seventh day after discharge showed CRP 0.07 mg/dL and WBC 11620/μL (Eosino 27.0%), so outpatient follow-up was terminated. Regarding Kimura's disease, steroids were not administered because of the history of necrosis of the femoral head and because the patient was infected.

## Discussion

This case was caused by SDSE bacteremia. The entry site for bacteremia was thought to be caused by head and neck skin and soft tissue infections associated with Kimura's disease. SDSE is a hemolytic streptococcus classified C and G according to the Lancefield classification. This bacterium causes pharyngitis and skin/soft tissue infections [5]. It has been reported to occur more often in patients with underlying severe diseases [6]. Risk factors include older age, diabetes, and malignant tumors, and local factors include physical and functional disorders of skin and soft tissues such as lymphedema and skin lesions [6]. This bacterium has been reported to have C and G antigens and SDSE, which has acquired A antigen [7]. This is the reason why the GAS rapid antigen test, in this case, was positive.

The patient had a history of incision and drainage for a left thyroid abscess at another hospital three years ago. Streptococcus constellatus, a hemolytic streptococcus, was detected in an abscess at the same site. This streptococcus has been the subject of taxonomic confusion, with no clear Lancefield classification. The representative groups are A, C, F, and G; the rest are unclassifiable [8]. Streptococcus constellatus is often associated with dental plaque and periodontal disease, but its association with skin and soft tissue diseases has also been reported [9-11].

The organs infected by group G hemolytic streptococcus (GGS) infections account for 59.6% cellulitis, 19.1% primary bacteremia, and 4.3% skin and soft tissue infections, accounting for 83%. Furthermore, GGS bacteremia has a high recurrence rate, and chronic local lymph abnormalities and blood circulation abnormalities due to malignant tumors, trauma, lymph node dissection, etc., are thought to be one of the causes. It has been reported that the incidence of recurrent cellulitis with bacteremia is 14.1 times higher in patients with lymphatic abnormalities [12]. This case had a history of hospitalization for streptococcal infection and bacteremia four years ago and was hospitalized for the same reason this time as well. The causative bacteria were streptococcal infections associated with cellulitis and skin and soft tissues. This may be related to the physical and functional disorders of the skin and soft tissues of Kimura's lymphoproliferative disease. It has been reported that Streptococcus constellatus reacts quickly with A, C, F, and G antiserum [8]. The common denominator between SDSE and Streptococcus constellatus is that they respond to G antigen [5,8]. For this reason, when Kimura’s disease is complicated by streptococcal infection, it is reasonable to consider GGS, which has a higher recurrence rate than the typical GAS.

## Conclusions

A proliferation of lymphoid follicles accompanies Kimura's disease. There is a relationship between lymphoid follicle proliferation and streptococcal infection. If a patient with a history of Kimura disease exhibits fever and a high inflammatory response, it is important to rule out streptococcal infection.
